# Using a health-rating system to evaluate the usefulness of *Caenorhabditis elegans* as a model for anthelmintic study

**DOI:** 10.1371/journal.pone.0179376

**Published:** 2017-06-20

**Authors:** Kathryn J. Weaver, Cassandra J. May, Brian L. Ellis

**Affiliations:** Department of Biology and Chemistry, Bethel College, Mishawaka, Indiana, United States of America; Duke University School of Medicine, UNITED STATES

## Abstract

Soil-transmitted helminths (STHs) are intestinal parasitic nematodes that infect humans, and are transmitted through contaminated soil. These nematodes include the large roundworm (*Ascaris lumbricoides*), whipworm (*Trichuris trichiura*), and hookworm (*Ancylostoma ceylanicum*, *Ancylostoma duodenale*, and *Necator americanus*). Nearly 1.5 billion people (~24% of the population) worldwide are infected with at least one species of these parasites, burdening the poor, in particular, children and pregnant women. To combat these diseases, the WHO only recognizes four anthelmintic drugs, including the preferred drug, albendazole, for mass drug administration (MDA). These four drugs have a total of two different mechanisms of action, and, as expected, resistance has been observed. This problem calls for new drugs with different mechanisms of action. Although there is precedence for the use of *Caenorhabditis elegans* (*C*. *elegans*), a free-living nematode, as a model for drug screening and anthelmintic testing, their usefulness for such anthelmintic study is not clear as past research has shown that *C*. *elegans* did not show a strong response to albendazole, the MDA drug of choice, in comparison with various STHs under similar treatment. To further examine if *C*. *elegans* has the potential to be a good model organism for anthelmintic drug study, we employed a health rating scale in order to tease out potential effects of albendazole, and other anthelmintics, that may have been missed using a binary, dead/alive scale. Using the health-rating scale we found that although the worms may have not been dying, they were sick, showing dose responses to anthelmintic drugs, including albendazole, reinforcing *C*. *elegans* as a useful model for anthelmintic study.

## Introduction

About 1.5 billion people in the world are infected with soil-transmitted helminths (STHs) and most of these people are the poorest people in the world [1]. The infections have devastating effects on human growth, nutrition, cognition, school attendance/performance, pregnancy, work production and earnings. Those infected with soil-transmitted helminthes are among the poorest people in the world in part because of the parasite trap, that is, that impoverished people are more likely to have worms because of their unsanitary living conditions and the negative effects on work production and earnings often keep them in poverty [2–4]. Even those who are not infected (or have been dewormed) but live in poverty are more likely to be infected due to unsanitary living conditions moving them into the parasite trap. Furthermore, soil-transmitted helminth infections are co-endemic with and have been shown to negatively affect HIV, malaria, and tuberculosis [5–9]. Currently, the WHO recognizes four drugs, falling into two classes: nicotinic acetylcholine receptor agonists (pyrantel and levamisole) and benzimidazoles (albendazole and mebendazole) for Mass Drug Administration. In practice, only one drug, albendazole, is mainly used in mass drug administration (MDA) [10–11]. Alternate drug used for treatment of filarial nematode infections in humans, but not typically for STHs due to its poor efficacy in treating whipworm and hookworm, is the macrocyclic lactone in the avermectin drug family, ivermectin [11–12]. Ivermectin activates glutamate-gated chloride channels leading to nerve and muscle hyperpolarization and resulting worm paralysis [13–15]. The 2015 Nobel Prize in Physiology/Medicine was awarded to the discoverers of artemisinin and ivermectin since ivermectin has been used to successfully treat lymphatic filariasis (elephantiasis) and Onchocerciasis (river blindness) [16]. Nitazoxanide, a thiazolide, is another drug that has been used in treating STHs given to patients in six doses for three days [17]. Nitazoxanide was discovered in 1984 because of its efficacy against tapeworms [18]. Nitazoxanide was found to be effective in treatments against various bacterial infections and as an antiviral agent [19]. Rather surprisingly, nitazoxanide was recently found to be effective in treating STHs [20–23]. The primary drug used for MDA is albendazole because of its low cost of production and wide efficacy against microfilariae and adult helminths. It is thought that albendazole inhibits the assembly of microtubules, decreasing the uptake of glucose and synthesis of ATP [24]. As is expected for any chemotherapeutic, resistance to this drug is already emerging [25–29]. This problem calls for new drugs with different mechanisms of action. Incredibly, all of the current drugs administered for the treatment of soil-transmitted helminth infections were not originally developed in answer to human infections, but rather for the market in veterinary use [30–31] and so it is likely that the very best drugs for human parasites have yet to be discovered.

Sadly, because the people mostly affected by these STHs are the most impoverished in the world, very little money has gone into drug discovery and design since, as estimated by Tufts in 2014, the process of discovery, design, and clinical trials costs 2.6 billion dollars [32]. Thus these diseases have been termed Neglected Tropical Diseases (NTD) by the WHO and Peter Hotez. Even in academia the amount of money that goes toward anthelmintic research focused on the most important STHs is abysmal. According to the 2015 G-Finder, in 2014 over a billion dollars was spent on HIV/AIDS and a little over 600 million on Malaria, while only 97.3 million dollars was spent on Helminth R&D [33]. The NIH lists funding in 2014 for cancer to be upwards of 5 billion dollars, aging research nearly 2.5 billion, diabetes research near a billion dollars, asthma around 200 million dollars, and Alzheimer’s around 500 million dollars [34]. The comparatively low funding level is contrast to the disease burden STHs cause, as disease due to these STHs is third behind HIV/AIDS and malaria in Years Lost due to Disability (YLD) but far more prevalent than both [35]. The WHO describes YLD in this way, “YLD is the years lost due to disability for people living with the health condition or its consequences” and is calculated by the product of the number of incident cases, disability weight, and the average duration of the case until remission or death in years [36].

The low amount of funding translates into a low amount of laboratories that can afford to do anthelmintic study. Although multiple NGOs, such as the Bill and Melinda Gates foundation, have begun to step in and donate their resources and bring attention to this problem, it is obvious more funding and attention is needed. Furthermore, the way in which the money is utilized, that has been directed towards anthelmintic study, needs to be constantly reviewed to ensure it is spent in the most efficient and productive ways.

One of the most cost-effective ways to screen for new drugs and to further develop and study anthelmintics is to utilize the free-living nematode *Caenorhabditis elegans*. This roundworm is a powerful model organism that is well characterized and cost-effective and has been used in the past as a readily available laboratory model for studying veterinary anthelmintics [37–41]. However, it is not clear that it is a good model for studying and identifying anthelmintic drugs. For example, in a comprehensive comparison of the effects of all major classes of anthelmintics on major STHs of humans, related species, and one non-parasitic nematode (Hu *et al*., 2013) showed that *C*. *elegans* did not respond to stratified treatment with albendazole, whereas hookworms, whipworms, and Ascaris under similar treatment were negatively impacted by at least one concentration [37]. This is significant because albendazole is considered the drug of choice for treatment in humans, and ideally a model for studying anthelmintics should be similarly affected. In contrast, a review of anthelmintic drugs by Holden-Dye and Walker (2014) concluded that in regard to *C*. *elegans* as a model parasite, the free-living nematode was not appropriate for studying questions of parasitic life cycle; however, they also concluded that the free-living nematode is an appropriate model for studying comparative physiology and pharmacology for the phylum Nematoda [38,39]. These conflicting conclusions require further investigation concerning the potential of *C*. *elegans* as a model parasite in anthelmintic discovery and study.

In this study, to further investigate if *C*. *elegans* has potential to be a good model for anthelmintic study, we utilized the health rating system employed by Hu *et al*. (used for scoring parasites) to determine if we could tease out anthelmintic effects from drugs that are missed in a binary, dead/alive, rating system. We also compare the binary dead/alive results (derived from the health rating results) to previous work [37] to validate the use of a health rating scale. Drugs selected for determining if *C*. *elegans* is a good model for anthelmintic study were the following: albendazole (benzimidzole), pyrantel (nAChR agonist), ivermectin (macrocyclic lactone), and nitazoxanide (thiazolide); four clinically-used drugs comprising all the major classes of anthelmintics. L4 stage *C*. *elegans*, were treated with these drugs as this species has been suggested and used for anthelmintic screening [37–39]. Intoxication was scored using a relative health system (motility index score) which was then translated into motility (dead-alive) for comparison with past work. In evaluating anthelmintic efficacy against nematodes *in vitro*, motility has been the most common method used [37, 42–45]. This anthelmintic inhibition of nematode motility is used as a standard because *in vivo* anthelmintic paralysis of STHs is thought to play an important role in parasite clearance [46]. Previous studies have found that, with the exception of ivermectin, anthelmintic drugs have less efficacy on *C*. *elegans* than on parasites when scored with the dead-alive scale. It is thought that this decreased efficacy is due to the relative impermeability of the *C*. *elegans* cuticle [37, 47].

Within these health rating results, to truly be a good option in drug screening and study, *C*. *elegans* must demonstrate a dose-dependent response, specifically to albendazole, but also, ideally, to other anthelmintic drugs like pyrantel, ivermectin, and nitazoxanide. Finally, to determine the variability of drug efficacy in a single species, we tested the effects of albendazole on the Hawaiian strain (CB4856) of *C*. *elegans* and compared the efficacy with that of albendazole on Bristol N2 *C*. *elegans* since compared to other *C*. *elegans* wild isolates, the Hawaiian strain is considered to exhibit the highest genetic divergence from the N2 strain [48]. Here, we report that the Bristol N2 *C*. *elegans* do respond in a dose dependent manner to albendazole, ivermectin, pyrantel, and nitazoxanide. Furthermore, we find that the Hawaii *C*. *elegans* strain (CB4856) is more sensitive to albendazole when compared to the Bristol N2 strain.

## Materials and methods

### Ethics statement

Because unregulated animals were used in this study, ethics approval was not required.

### Nematode maintenance

*C*. *elegans* wild-type strain N2 Bristol and wild-isolate strain Hawaii (CB4856) were maintained on Nematode growth (NG) plates with *Escherichia coli* (*E*. *coli*) strain OP50 as food [49]. *C*. *elegans* age was synchronized by chunking NG plates with starved *C*. *elegans* onto OP50 seeded Enriched Nematode Growth (ENG) plates, bleaching the gravid adults according to the documented process after three days, and seeding synchronized first stage larvae (L1) onto OP50 seeded fresh ENG plates 44 hours before setting up an assay [50]. The *C*. *elegans* strains (N2 Bristol and Hawaiian CB4856) and *E*. *coli* OP50 were generous gifts from the Aroian Lab at University of Massachusetts Medical School.

### Reagents

Reagents used for maintenance and experimentation have been previously described [50, 51]. Most of the chemicals that were used in this study including NaCl (catalog no. BDH8014), KH_2_PO_4_ (catalog no. P5379), K-citrate (catalog no. P1722), EDTA (catalog no. 1233508), FeSO_4_ (catalog no. 215422), MnCl_2_ (catalog no. 244589), ZnSO_4_ (catalog no. 204986), CuSO_4_ (catalog no. 451657), MgSO_4_ (catalog no. M7506), and cholesterol (catalog no. C8667) were purchased from Sigma-Aldrich; ethanol (catalog no. 861300) was bought from Carolina; and K_2_HPO_4_ (catalog no. 7092) was purchased from Mallinckrodt.

Four drugs were utilized in this study: albendazole (Sigma-Aldrich, catalog no. A4673), ivermectin (Sigma-Aldrich, catalog no. I8898), nitazoxanide, which was kindly provided by Romark laboratories, and pyrantel pamoate (Sigma Aldrich, catalog no. P6210) in place of pyrantel tartrate (catalog no. P7674) which was used by Hu *et al*.; however, at the time of this study, pyrantel tartrate was no longer sold by Sigma-Aldrich. Pyrantel pamoate is used both for human treatment and veterinary medicine. 10 mg of fresh albendazole, pyrantel pamoate, ivermectin, or nitazoxanide were dissolved in 20 μL 100% DMSO (Sigma-Aldrich, catalog no. D8418) and subsequently diluted with 180 μL deionized water (reaching a stock concentration of 50 mg/mL in 10% DMSO). Serial dilutions (10-fold) were completed with 180 μL of freshly made stock 10% DMSO (90% deionized water) and 20 μL of drug from the next highest concentration. By adding the drug to the wells (10 μL for a final volume of 500 μL in each well), the drug in DMSO was diluted another fifty-fold, bringing the final concentration of DMSO to 0.2% in all the wells. Every concentration (0.1, 1, 10, 100, and 1000 μg/mL), including the control (10 μL of 10% DMSO but not drug), was tested in triplicate for each assay.

### *In vitro* assays

Using 24-well Polystyrene individually packaged Non-Tissue Culture Treated Plate (Falcon 351147), approximately 5–20 synchronized fourth-stage larvae (L4) *C*. *elegans* were added to 500 μL of special S Medium, OD = 3.0 OP50, and 8mM 5-Fluoro-2’-deoxyuridine (FudR) (Sigma-Aldrich, catalog no. 343333) cocktail and varying concentrations of anthelmintic drugs in triplicate. FudR is included in the wells to inhibit the production of viable eggs [52]. Well- plates were wrapped in damp paper towel to prevent evaporation and incubated at 25°C. Worm motility was scored daily on a 3-2-1-0 scale for seven days. “3” represents a worm with vigorous movement similar to control with no drug; “2” represents a worm with whole-body movements (seen without external stimulus) visibly slower than control; “1” represents a worm that was not moving on its own but moved when touched with an eyelash pick 3 times over the course of a few seconds; “0” represents a worm that did not move even when prodded 3 times [37]. Each assay was performed at least three times independently. Assays with controls exhibiting less than an 80% survival rate were not included in the overall average. The total number of nematodes treated by drug and concentration are reported in Table 1.

**Table 1:** Number of nematodes treated.

	PYR	NTZ	IVM	ALB (N2)	ALB (Hawaii)
0 μg/mL	247	69	78	70	127
0.1 μg/mL	228	63	72	100	141
1 μg/mL	176	61	71	104	144
10 μg/mL	188	75	75	101	135
100 μg/mL	186	58	76	91	148
1000 μg/mL	140	54	51	116	144

### Data analyses

Worm survival was calculated on a 1–0 scale. “1” represents a worm that was either moving without stimulus, or moved after a stimulus with an eyelash pick by touching the worm 3 times over the course of a few seconds. “0” represents a worm that did not move, even after stimulus with an eyelash pick, touching the worm 3 times, over the course of a few seconds. We note that the most severe phenotype we scored was the failure of the nematodes to move when prodded. This lack of movement and complete paralysis was scored as a 0 (for both the health rating scale and the dead-alive scale) and the nematode was considered dead. However, we did not test for recovery of these nematodes in a media absent of drug after the completion of each day scoring and therefore cannot be certain that such nematodes, were, in fact, dead (instead of completely paralyzed); the phenotype of complete paralysis of nematodes has been used as a standard in anthelmintic studies [37, 42–45]. The triplicate wells from single assays were combined to form one large experiment and then averaged. This produced an average value for each drug concentration and day combination. Since assays were initially scored on a health rating scale as described earlier, a corresponding dead-alive score was obtained by combining the number of worms with a score of 3, 2, or 1 and calling them alive (thus a “1”). The number of worms with a “0” score in the health-rating system is the same for the number of worms in the 1–0 scoring (thus a “0”) and were called dead. These average values were graphed with GraphPad Prism version 7.02 for Windows (GraphPad Software, La Jolla, CA, USA) to show representative motility index score and percent survival. We note that potential subjectivity could be introduced using the health-rating scale, in particular discerning a “3” from a “2”. To evaluate the extent of subjectivity, we had 4 different people score multiple albendazole experiments individually and found that the data from each person was nearly identical.

IC_50_, LT_50_, and IT_50_ values were also calculated with GraphPad Prism version 7.02 for Windows. We selected day 4 (mid-point of the 7-day experiment) as the day to calculate and report IC_50_ values (concentration where 50% of *C*. *elegans* are inhibited). Thus, a logarithmic transformation followed by a nonlinear fit were performed on day 4 data. For 1–0 scoring, inhibited was defined to be a score of 0 as previous work had done [37]. For 3–0 scoring, inhibited was defined to be a score of 2, 1, or 0. For LT_50_ values (the day where 50% of *C*. *elegans* are dead, score 0, at a given concentration), survival curves were constructed with the event being defined as death (score 0). Similarly, corresponding to LT_50_ values, for IT_50_ values (the day where 50% of *C*. *elegans* are inhibited at a given concentration), survival curves were constructed with the event being defined as inhibition (score 2, 1, or 0). We note that a score of 0 was included in the category of “inhibited” as previous work also included it for IC_50_ tests [37]. A graphical representation of these IC_50_, LT_50_, and IT_50_ values presented in the tables can be found in supporting information (S1, S2, S3, S4 and S5 Figs).

Chi-square test was performed using Microsoft Excel 2016 and R to compare the day 7 non-drug-induced death to drug-induced death to survival of Bristol N2 and Hawaii (CB4856) strains treated with 1000 μg/mL albendazole.

## Results

### Effects of pyrantel on N2 strain: 1–0 scale vs. 3–0 scale

**Fig 1:**
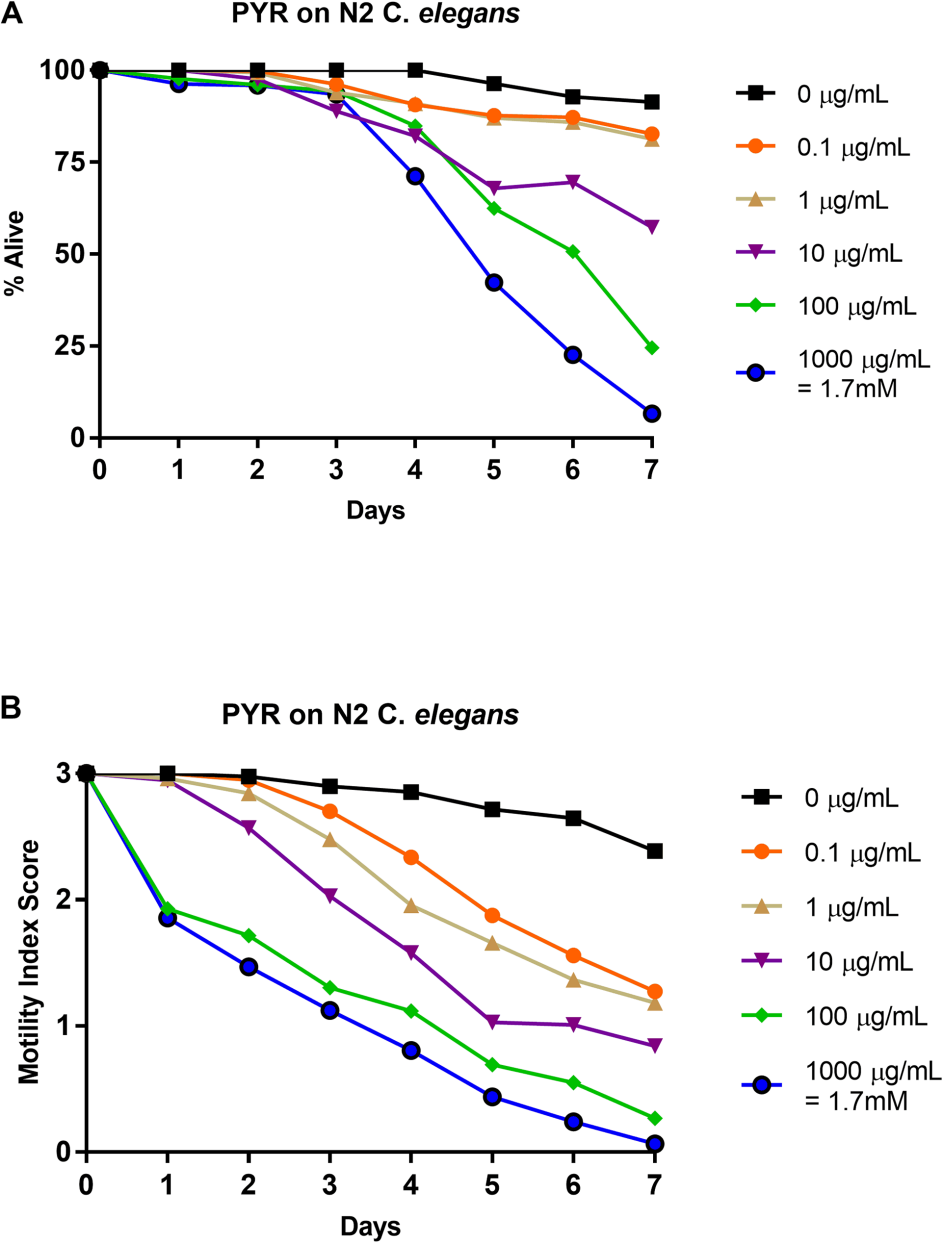
Average intoxication of N2 *C*. *elegans* (L4 stage) using five doses of pyrantel pamoate (PYR). **a). Dead-Alive (1–0) scale. b). Health rating (3–0) scale.** For comparative reference, the molar concentrations of each drug at 1000 μg/mL is indicated. Roundworms were scored daily for 7 days for motility. A score of 3 represented a worm with vigorous movement. A score of 2 represented a worm with whole body movements without external stimulus, but visibly slower than the control. A score of 1 represented a worm with movement only after the introduction of external stimulus. A score of 0 represented a worm with no movement, even after the introduction of external stimulus. To translate these scores into the binary dead or alive, a score of 3, 2, or 1 was considered a score of 1 (alive) and a score of 0 was considered a score of 0 (dead). Data are the combination of at least three independent trials.

N2 *C*. *elegans* L4-staged were subjected to five doses of pyrantel pamoate (0.0 μg/mL, 0.1 μg/mL, 1 μg/mL, 10 μg/mL, 100 μg/mL, and 1000 μg/mL) for seven days. Each day, relative health, based on a motility index score (3–0), was observed and recorded (Fig 1B) and then the motility index score results were translated into a basic motility score (1–0) (Fig 1A) in order to compare them to the work of *Hu et al*. [37] and to highlight the differences in scoring format. Based on the basic motility score, in this study, pyrantel showed a higher efficacy at the three highest concentrations than previous studies had documented, with survival below 10% at the highest concentration [37]. Noticeable separation in efficacy occurred day 4 as opposed to day 1 in previous studies [37], but the responses to the lower doses were similar to those previously reported showing survival above 75% [37] (Fig 1A). LT_50_ values were undefined for concentrations below 10 μg/mL and defined as day 7 for 10 μg/mL, day 6 for 100 μg/mL, and day 5 for 1000 μg/mL (Table 2). The IC_50_ value on day 4 for 1–0 scoring (Table 3) was greater than 1000 μg/mL and this is greater than the previously reported 746.86 μg/mL [37]. When utilizing the 3–0 rating system, negative effects were observed early (day 1), with a steady decline in overall health of the nematodes at all concentrations in a stratified manner (Fig 1B). The IC_50_ value on day 4 for 3–0 scoring (Table 3) was 0.375 μg/mL. As opposed to LT_50_ values, which were defined at higher concentrations only, IT_50_ values were defined at the lowest drug concentrations as day 4 for both 0.1 and 1 μg/mL, day 3 for 10 μg/mL, and day 1 for both 100 and 1000 μg/mL (Table 2). See S1 Fig for graphical representation of these values.

**Table 2:** LT_50_ and IT_50_ values (days).

	**Pyrantel**											
	**0 μg/mL**		**0.1 μg/mL**		**1 μg/mL**		**10 μg/mL**		**100 μg/mL**		**1000 μg/mL**	
	***LT***_***50***_	***IT***_***50***_	***LT***_***50***_	***IT***_***50***_	***LT***_***50***_	***IT***_***50***_	***LT***_***50***_	***IT***_***50***_	***LT***_***50***_	***IT***_***50***_	***LT***_***50***_	***IT***_***50***_
**Bristol N2*C*. *elegans***	**U**	**U**	**U**	**4**	**U**	**4**	**7**	**3**	**6**	**1**	**5**	**1**
	**Nitazoxanide**											
	**0 μg/mL**		**0.1 μg/mL**		**1 μg/mL**		**10 μg/mL**		**100 μg/mL**		**1000 μg/mL**	
	***LT***_***50***_	***IT***_***50***_	***LT***_***50***_	***IT***_***50***_	***LT***_***50***_	***IT***_***50***_	***LT***_***50***_	***IT***_***50***_	***LT***_***50***_	***IT***_***50***_	***LT***_***50***_	***IT***_***50***_
**Bristol N2*C*. *elegans***	**U**	**U**	**U**	**3**	**U**	**3**	**U**	**3**	**U**	**3**	**5**	**2**
	**Ivermectin**											
	**0 μg/mL**		**0.1 μg/mL**		**1 μg/mL**		**10 μg/mL**		**100 μg/mL**		**1000 μg/mL**	
	***LT***_***50***_	***IT***_***50***_	***LT***_***50***_	***IT***_***50***_	***LT***_***50***_	***IT***_***50***_	***LT***_***50***_	***IT***_***50***_	***LT***_***50***_	***IT***_***50***_	***LT***_***50***_	***IT***_***50***_
**Bristol N2*C*. *elegans***	**U**	**U**	**U**	**2**	**7**	**1**	**6**	**1**	**5**	**1**	**5.5**	**1**
	**Albendazole**											
	**0 μg/mL**		**0.1 μg/mL**		**1 μg/mL**		**10 μg/mL**		**100 μg/mL**		**1000 μg/mL**	
	***LT***_***50***_	***IT***_***50***_	***LT***_***50***_	***IT***_***50***_	***LT***_***50***_	***IT***_***50***_	***LT***_***50***_	***IT***_***50***_	***LT***_***50***_	***IT***_***50***_	***LT***_***50***_	***IT***_***50***_
**Bristol N2*C*. *elegans***	**U**	**U**	**U**	**5**	**U**	**4**	**U**	**4**	**U**	**3**	**U**	**3**
**Hawaii *C*. *elegans***	**U**	**U**	**U**	**4**	**U**	**4**	**U**	**2**	**U**	**2**	**U**	**2**

U = undefined

**Table 3:** IC_50_ values (μg/mL) at day 4.

	Hu et al IC_50_ results	1–0 Scoring IC_50_	3–0 Scoring IC_50_
**PYR (N2)**	**746.86**	**> 1000**	**0.375**
**NTZ (N2)**	**> 1000**	**> 1000**	**< 0.1**
**IVM (N2)**	**> 1000**	**> 1000**	**< 0.1**
**ALB (N2)**	**> 1000**	**> 1000**	**1.4**
**ALB (Hawaii)**	**No Data**	**> 1000**	**0.147**

### Effects of nitazoxanide on N2 strain: 1–0 scale vs. 3–0 scale

**Fig 2:**
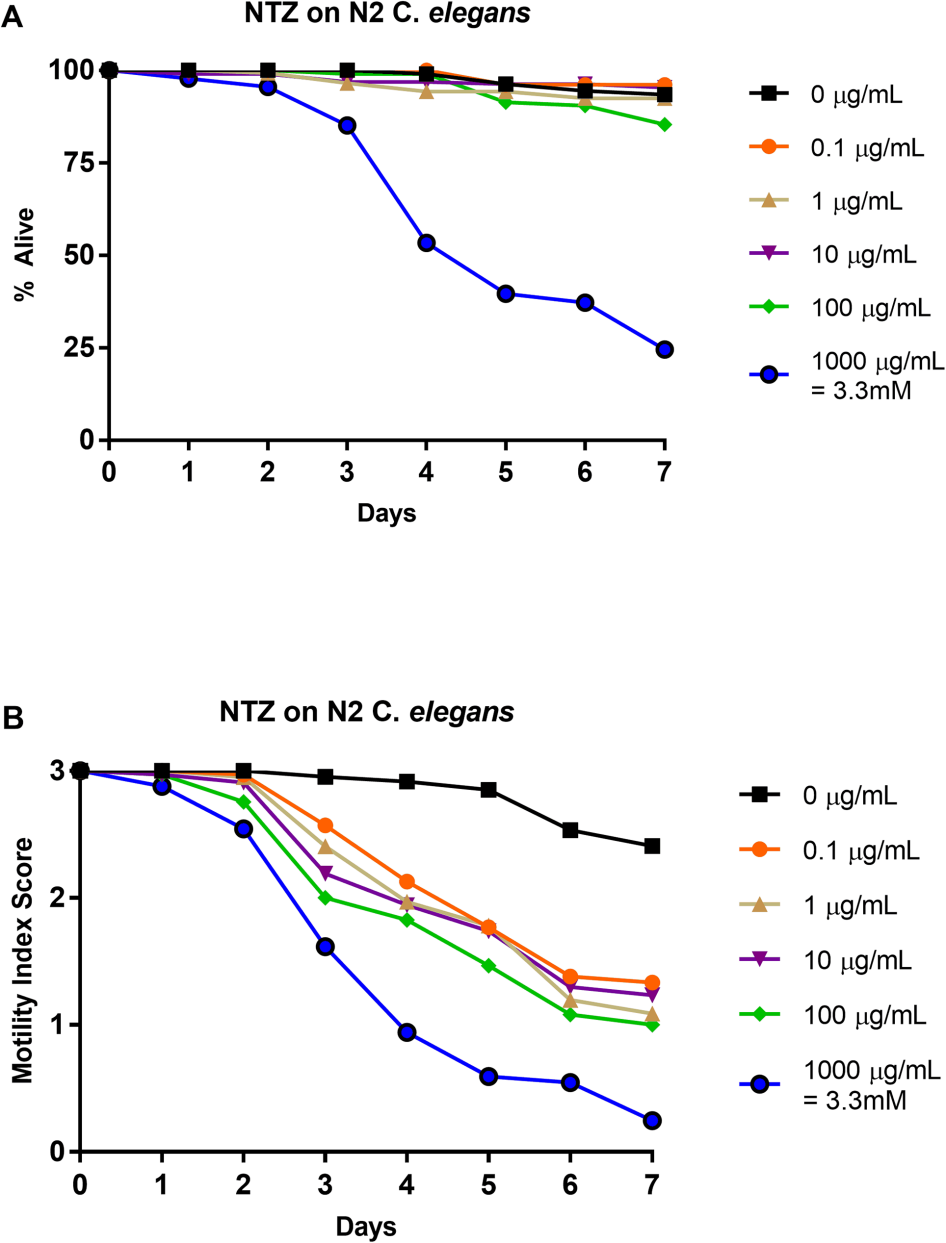
Average intoxication of N2 *C*. *elegans* (L4 stage) using five doses of nitazoxanide (NTZ). **a). Dead-Alive (1–0) scale. b). Health rating (3–0) scale.** For comparative reference, the molar concentrations of each drug at 1000 μg/mL is indicated. Roundworms were scored daily for 7 days for motility. A score of 3 represented a worm with vigorous movement. A score of 2 represented a worm with whole body movements without external stimulus, but visibly slower than the control. A score of 1 represented a worm with movement only after the introduction of external stimulus. A score of 0 represented a worm with no movement, even after the introduction of external stimulus. To translate these scores into the binary dead or alive, a score of 3, 2, or 1 was considered a score of 1 (alive) and a score of 0 was considered a score of 0 (dead). Data are the combination of at least three independent trials.

N2 *C*. *elegans* L4-staged were subjected to five doses of nitazoxanide (0.0 μg/mL, 0.1 μg/mL, 1 μg/mL, 10 μg/mL, 100 μg/mL, and 1000 μg/mL) for seven days. Each day the relative health, based on the motility index score (3–0) was observed and recorded (Fig 2B). The motility index score results were then translated into basic motility scores (1–0) (Fig 2A) in order to compare to previous work [37] and to highlight the differences in scoring format. When utilizing the basic motility score, nitazoxanide displayed a comparable efficacy to that observed in past studies [37]. At the highest concentration, lower than 25% survival was observed while all other concentrations showed survival above 75% (Fig 1A). The IC_50_ value at day 4 for 1–0 scoring (Table 3) was greater than 1000 μg/mL as had also been previously reported [37]. LT_50_ values were undefined at 0 μg/mL, 0.1 μg/mL, 1 μg/mL, 10 μg/mL, and 100 μg/mL, while the LT_50_ for 1000 μg/mL was defined to be day 5 (Table 2). The health-rating results (Fig 2B) showed an early and steady health decline at every concentration. The corresponding IC_50_ value at day 4 for 3–0 scoring (Table 3) was less than 0.1 μg/mL. In contrast to the undefined LT_50_ values at all concentrations, IT_50_ values were defined as day 3 for 0.1, 1, 10, and 100 μg/mL and as day 2 for 1000 μg/mL (Table 2). See S2 Fig for graphical representation of these values.

### Effects of ivermectin on N2 strain: 1–0 scale vs. 3–0 scale

**Fig 3:**
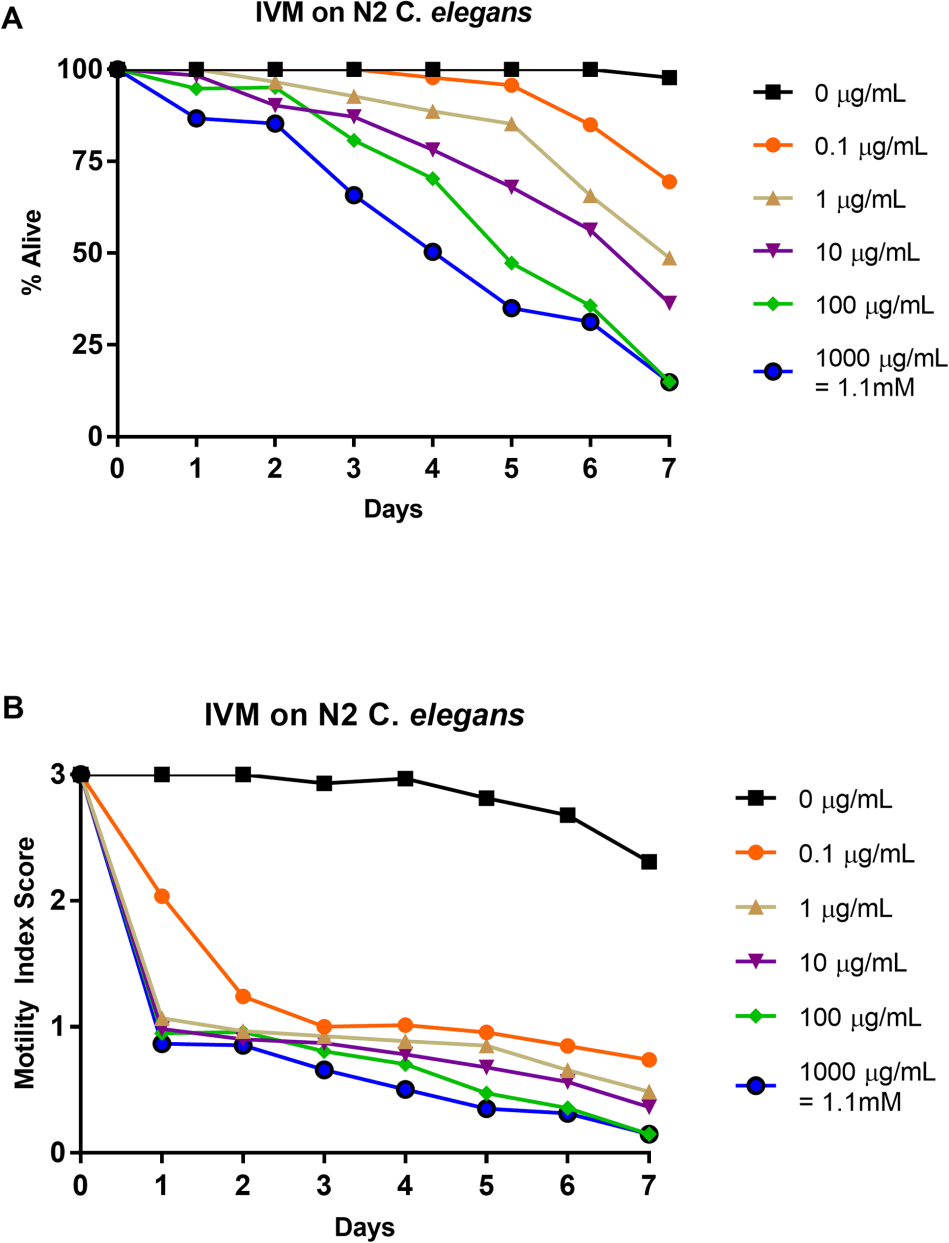
Average intoxication of N2 *C*. *elegans* (L4 stage) using five doses of ivermectin (IVM). **a). Dead-Alive (1–0) scale. b). Health rating (3–0) scale.** For comparative reference, the molar concentrations of each drug at 1000 μg/mL is indicated. Roundworms were scored daily for 7 days for motility. A score of 3 represented a worm with vigorous movement. A score of 2 represented a worm with whole body movements without external stimulus, but visibly slower than the control. A score of 1 represented a worm with movement only after the introduction of external stimulus. A score of 0 represented a worm with no movement, even after the introduction of external stimulus. To translate these scores into the binary dead or alive, a score of 3, 2, or 1 was considered a score of 1 (alive) and a score of 0 was considered a score of 0 (dead). Data are the combination of at least three independent trials.

N2 *C*. *elegans* L4-staged were subjected to five doses of ivermectin (0.0 μg/mL, 0.1 μg/mL, 1 μg/mL, 10 μg/mL, 100 μg/mL, and 1000 μg/mL) for seven days. Relative health was observed and recorded each day using a motility index score (3–0) (Fig 3B). This data was then translated into a basic motility score (1–0) (Fig 3A) in order to compare to previous work [37] and to highlight the differences in scoring format. The highest concentration caused a high percentage of complete paralysis while the lower concentrations caused paralysis at lower levels (Fig 3A). This paralysis appears to shrink the nematodes, which remain motionless unless an outside stimulus is introduced. Although many of the intoxicated worms are very small and obviously sick, many do move when prodded. These results were comparable to past work, however lower concentrations in the past showed a slightly lower percent survival [37] (Fig 3A). The IC_50_ value at day 4 for 1–0 scoring was greater than 1000 μg/mL, the same as the previously reported value [37] (Table 3). The LT_50_ values were undefined for 0.1 μg/mL and defined for the rest of the concentrations (Table 2) with day 7 for 1 μg/mL, day 6 for 10 μg/mL, day 5 for 100 μg/mL, and day 5.5 for 1000 μg/mL (the 5.5 score was due to arriving at 50% survival exactly on day 5 for 1000 μg/mL). The health rating results (Fig 3B) showed a sudden decrease in nematode health at all concentrations followed with steady and stratified further decrease in relative motility. Ivermectin shows the strongest efficacy of all of the anthelmintics tested due to the severe paralysis and shrinking of the nematodes until, in some cases, eventual death (i.e. no further response to outside stimulus). The IC_50_ value on day 4 for the 3–0 scoring was less than 0.1 μg/mL (Table 3). The IT_50_ value for 0.1 μg/mL was day 2, while day 1 was the IT_50_ value for 1, 10, 100, and 1000 μg/mL (Table 2). See S3 Fig for graphical representation of these values.

### Effects of albendazole on N2 strain: 1–0 scale vs. 3–0 scale

**Fig 4:**
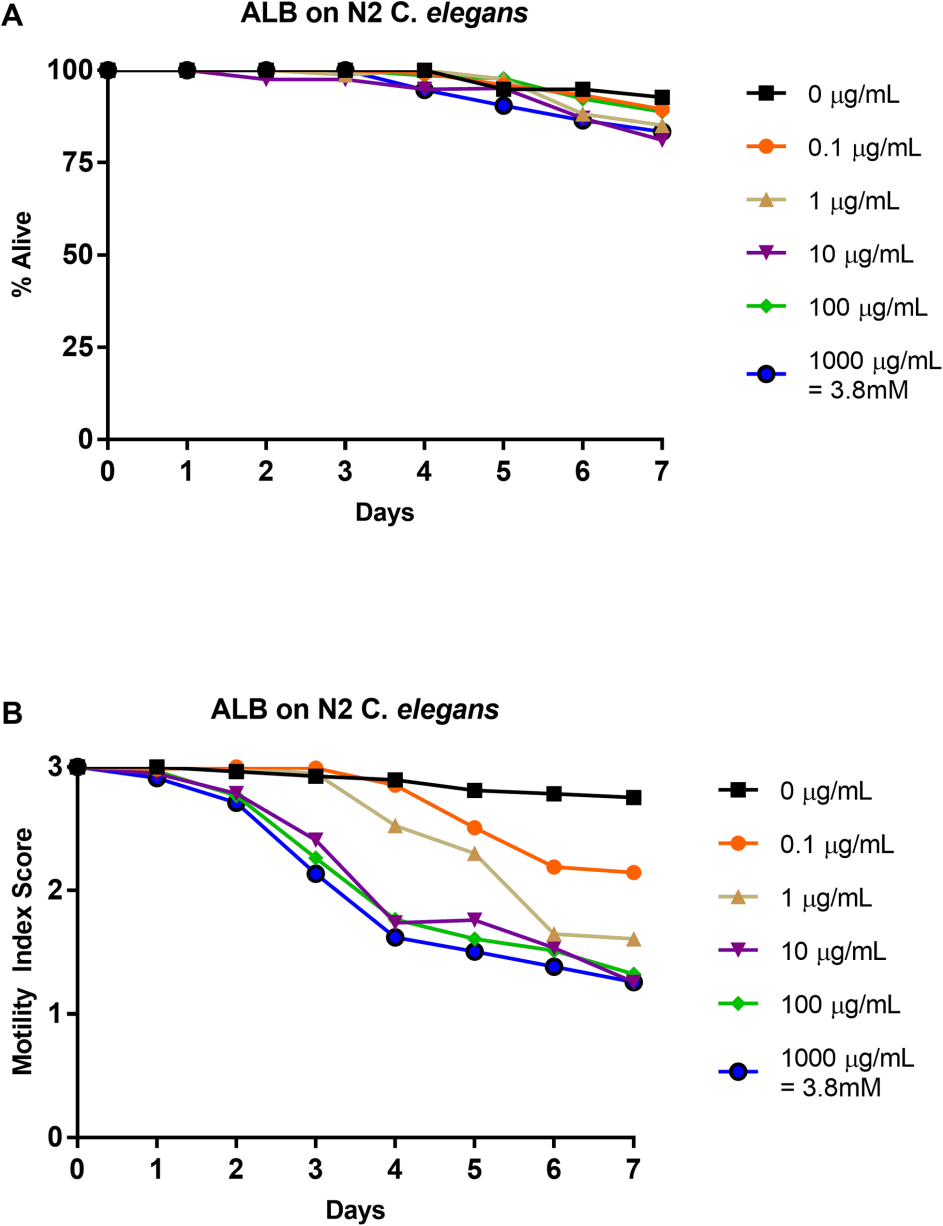
Average intoxication of N2 *C*. *elegans* (L4 stage) using five doses of albendazole (ALB). **a). Dead-Alive (1–0) scale. b). Health rating (3–0) scale.** For comparative reference, the molar concentrations of each drug at 1000 μg/mL is indicated. Roundworms were scored daily for 7 days for motility. A score of 3 represented a worm with vigorous movement. A score of 2 represented a worm with whole body movements without external stimulus, but visibly slower than the control. A score of 1 represented a worm with movement only after the introduction of external stimulus. A score of 0 represented a worm with no movement, even after the introduction of external stimulus. To translate these scores into the binary dead or alive, a score of 3, 2, or 1 was considered a score of 1 (alive) and a score of 0 was considered a score of 0 (dead). Data are the combination of at least three independent trials.

N2 *C*. *elegans* L4-staged were subjected to five doses of albendazole (0.0 μg/mL, 0.1 μg/mL, 1 μg/mL, 10 μg/mL, 100 μg/mL, and 1000 μg/mL) for seven days. Relative health was observed and recorded using a motility index score (3–0) (Fig 4B). These results were translated into a basic motility score (1–0) (Fig 4A) in order to compare them to previous studies [37] and to highlight the differences in scoring format. Similar to past work, the nematodes showed minimal response to albendazole with percent alive scores above 80% at all concentrations, including the control, for the duration of the experiment [37] (Fig 4B). The IC_50_ value at day 4 for 1–0 scoring was greater than 1000 μg/mL (Table 3), comparable to previous results [37]. LT_50_ values were undefined at all concentrations (Table 2). The health rating results show a mild decrease in nematode health as compared to the other anthelmintics, producing a shallower curve ending with motility index scores greater than 1 for all concentrations (Fig 4B). Yet, a steady decrease in health at all concentrations was observed, with the higher concentrations showing similar efficacy. Thus, in contrast to the dead-alive results, which showed no effect on the albendazole treated nematodes, the health-rating results show a definitive effect on the health of the albendazole treated nematodes (Fig 4A and 4B). The IC_50_ value at day 4 for 3–0 scoring was 1.4 μg/mL (Table 3). The IT_50_ values were defined to be day 5 for 0.1 μg/mL, day 4 for 1 and 10 μg/mL, and day 3 for 100 and 1000 μg/mL (Table 2). These values were in contrast with the undefined LT_50_ values at all concentrations. See S4 Fig for graphical representation of these values.

### Effects of albendazole on Hawaii strain: 1–0 scale vs. 3–0 scale

**Fig 5:**
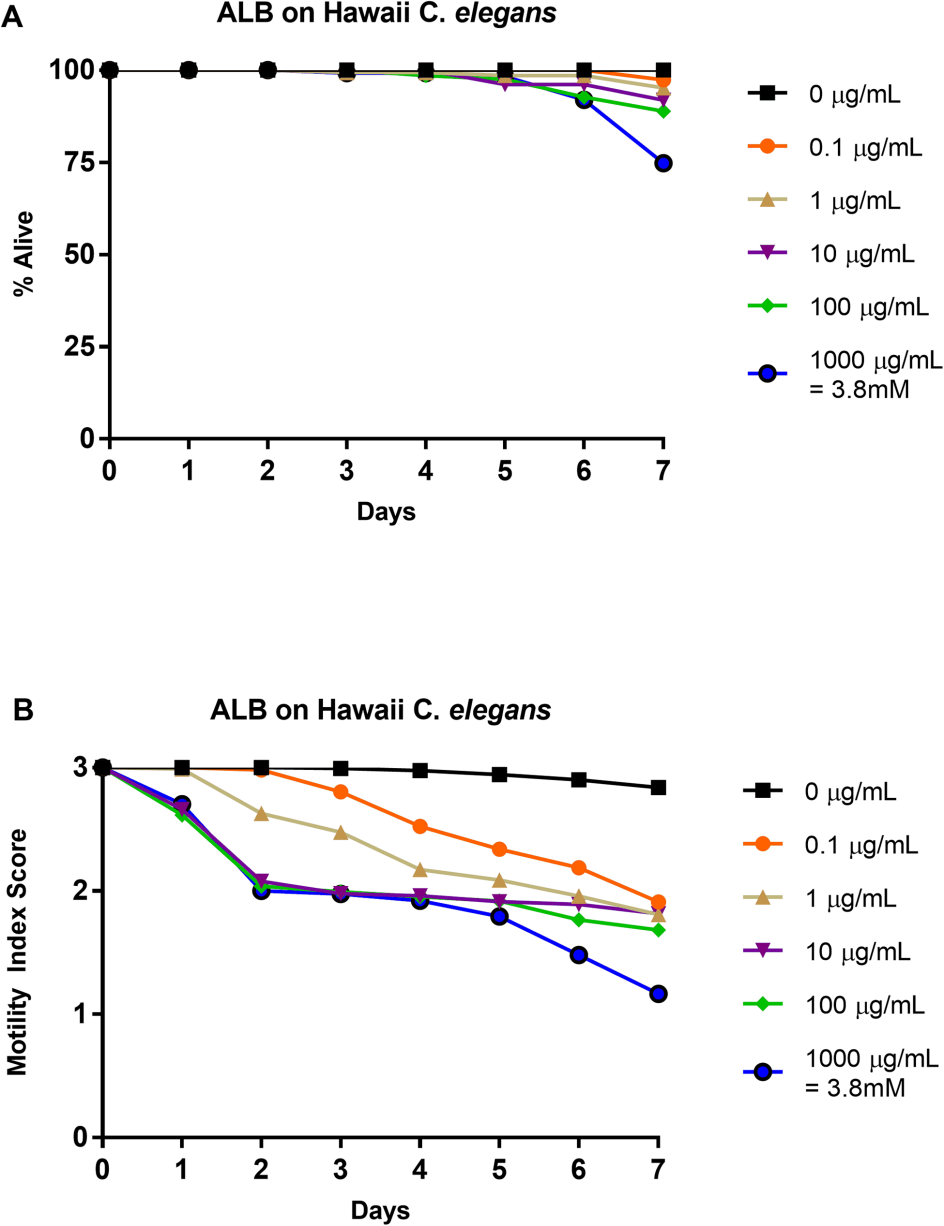
Average intoxication of Hawaii (CB4856) *C*. *elegans* (L4 stage) using five doses of albendazole (ALB). **a). Dead-Alive (1–0) scale. b). Health rating (3–0) scale.** For comparative reference, the molar concentrations of each drug at 1000 μg/mL is indicated. Roundworms were scored daily for 7 days for motility. A score of 3 represented a worm with vigorous movement. A score of 2 represented a worm with whole body movements without external stimulus, but visibly slower than the control. A score of 1 represented a worm with movement only after the introduction of external stimulus. A score of 0 represented a worm with no movement, even after the introduction of external stimulus. To translate these scores into the binary dead or alive, a score of 3, 2, or 1 was considered a score of 1 (alive) and a score of 0 was considered a score of 0 (dead). Data are the combination of at least three independent trials.

Hawaiian (CB4856) *C*. *elegans* L4-staged were subjected to five doses of albendazole (0.0 μg/mL, 0.1 μg/mL, 1 μg/mL, 10 μg/mL, 100 μg/mL, and 1000 μg/mL) for seven days. As an internal control, N2 worms were subjected to 0 μg/mL and 100 μg/mL. Relative health was observed and recorded using a motility index score (3–0) (Fig 5B). These results were translated into a basic motility score (1–0) (Fig 5A) in order to compare them to the N2 strain albendazole results. Percent survival of Hawaii *C*. *elegans* was similar to that of the N2 *C*. *elegans* treated with albendazole at all concentrations with an insignificant difference observed at 1000 μg/mL on day 7 (χ^2^ test, p = 0.15) when comparing drug-induced death to non-drug-induced death to survival (Fig 5B). Like the IC_50_ value for albendazole treated N2 *C*. *elegans*, the IC_50_ value on day 4 for 1–0 scoring of albendazole treated Hawaii *C*. *elegans* was greater than 1000 μg/mL (Table 3). Again, like the albendazole treated N2 *C*. *elegans*, the LT_50_ values were undefined at all concentrations (Table 2). Surprisingly, the IC_50_ value on day 4 for 3–0 scoring was 0.147 μg/mL, ten-fold less than the comparable value for albendazole treated N2 *C*. *elegans* (Table 3). IT_50_ values for 0.1 and 1 μg/mL were day 4, while for 10, 100, and 1000 μg/mL, the IT_50_ values were all day 2 (Table 2). These IT_50_ values occur earlier than the corresponding albendazole treated N2 *C*. *elegans* IT_50_ values, suggesting that Hawaii *C*. *elegans* are affected/inhibited sooner than N2 *C*. *elegans*. See S5 Fig for graphical representation of these values.

## Discussion

There are about 1.5 billion people infected with STHs worldwide. Those infected are typically the most impoverished people in the world. The WHO only recognizes 4 drugs for MDA, which only have 2 mechanisms of action, and resistance to the drugs has already been observed. New drugs, drug derivatives, and drug combinations all will be useful in the battling these infections. A robust model is needed to perform the discovery and testing. Ideally, human STHs would be used for this process but thus far only two human STHs can be maintained in immune competent animals (*Ancylostoma ceylanicum* and *Necator americanus* in hamsters) [53]. Next best would be STHs in animals that are closely related to human parasites; however, both of these strategies are costly both in terms of time and resources. Another option is to use the free-living nematode *C*. *elegans*. Although they are not STHs, there is precedence for using *C*. *elegans* in anthelmintic drug screening [37–41] because of its low cost and ease to maintain, but it is not entirely clear that the roundworm is a good model for studying and identifying anthelmintic drugs [37, 38, 41]. These conflicting conclusions require further investigation concerning the potential of *C*. *elegans* as a model parasite in anthelmintic study. In this study, to further investigate if *C*. *elegans* has potential to be a good model for anthelmintic study we utilized the health rating system employed by *Hu et al*. (used for scoring parasites) to determine if we could tease out anthelmintic effects from drugs that are missed in a binary dead/alive rating system. We also compare the binary dead/alive results (derived from the health rating results) to previous work [37] to validate the use of a health rating scale. Within these health rating results, to truly be a good option in drug screening and study, *C*. *elegans* must demonstrate a dose-dependent response, in general, but most importantly to albendazole, the drug of choice for MDA. Also to determine the variability of drug efficacy in a single species, we tested the effects of albendazole on the Hawaiian strain of *C*. *elegans* to compare the efficacy with that of albendazole on N2 *C*. *elegans*.

Overall, in comparison to previous data [37], our data matched up quite well. However, there were two observed dissimilarities evident from what was expected. *C*. *elegans* treated with 1000 μg/mL of pyrantel pamoate experienced a delayed but similar effect (ending % alive) as those previously treated with pyrantel tartrate [37]. Also, an increase in efficacy on *C*. *elegans* treated with both 10 μg/mL and 100 μg/mL of pyrantel pamoate was observed compared to those previously treated with pyrantel tartrate [37]; these differences are likely due to the use of the pyrantel pamoate instead of the use of pyrantel tartrate, which is no longer available from Sigma Aldrich. Although, this somewhat diminishes the comparison that can be made to the Hu et al. paper, it is beneficial in that pyrantel pamoate is less toxic to mice than pyrantel tartrate, likely due to solubility (personal communication with Yan Hu). Besides this disparity, the agreement between our dead-alive data and the data from Hu et al. (2013) suggests that our health-rating results properly translate to the accepted dead-alive results expected in *C*. *elegans* anthelmintic studies [37].

Through use of a health rating scale instead of a basic motility scale, several trends become evident when analyzing the data. First, dose-dependent responses were observed for each drug. Further, ivermectin produced the steepest inhibition over nearly all concentrations (Fig 3B). Over time, pyrantel and nitazoxanide displayed similar levels of inhibition as compared to ivermectin (Figs 1B and 2B respectively). The inhibition in *C*. *elegans* treated with albendazole was not as severe as *C*. *elegans* treated with ivermectin, pyrantel, or nitazoxanide, but importantly, a dose-dependent response showing decreased health was observed (Fig 4B).

The use of a health rating scale and the data analyzed with IC_50_ (Table 3) and IT_50_ (Table 2) calculations further showed the efficacy of the anthelmintic drugs to inhibit *C*. *elegans*. IT_50_ results further support that ivermectin has the greatest efficacy against *C*. *elegans*, with day 2 as the time for 50% of the nematodes to be inhibited at the lowest concentration while for other drugs at the same concentration, 50% inhibition is not reached until day 3 or 4 (Table 2). All IT_50_ values were defined at all concentrations while corresponding LT_50_ values were either undefined or occurred later (Table 2) confirming that a health rating scale gives a more comprehensive view of anthelmintic efficacy than a dead-alive scoring system when studying inhibition.

The treatment of Hawaii (CB4856) *C*. *elegans* with albendazole was compared to the treatment of Bristol N2 *C*. *elegans* with albendazole. A Chi-square test on 1000 μg/mL day 7 percent survival data showed that a greater number of Hawaii *C*. *elegans* died compared to N2 *C*. *elegans* was not significantly different statistically speaking. However, we cannot rule out a biological difference leading to this apparent difference in efficacy that suggests that at the highest concentration, albendazole has a somewhat greater efficacy against Hawaii *C*. *elegans* than against N2 *C*. *elegans*. Further, the coupling of the health rating results (Fig 5B) and the IC_50_ (Table 3) and IT_50_ (Table 2) values show that the difference in albendazole concentration needed to inhibit 50% of *C*. *elegans* was ten-fold different for the two strains, with the lower concentration inhibiting 50% of the Hawaii *C*. *elegans*. Also, for 0.1, 10, 100, and 1000 μg/mL, 50% of the Hawaii *C*. *elegans* were inhibited sooner (day 4, day 2, day 2, and day 2 respectively) than the N2 *C*. *elegans* at the same concentrations (day 5, day 4, day 3, and day 3 respectively). This data suggests that Hawaii *C*. *elegans* are more sensitive to albendazole than N2 *C*. *elegans*, and further work should explore the biological cause of this increased sensitivity. Furthermore, although the increased sensitivity was only tested and observed with albendazole, and requires further study with other anthelmintics, the data suggests that future work using *C*. *elegans* as a model for anthelmintic drug study should potentially switch from the N2 strain to the Hawaii strain. This study suggests that future anthelmintic study can use *C*. *elegans* and that when doing so it is beneficial to utilize a health-rating system, whether it be the one presented here or some other type of system, for example culturing *C*. *elegans* in CeHR axenic liquid medium and counting the replicated worm number [54]. For drug screening/discovery, however, we cannot recommend this particular system because utilizing an eyelash pick for stimulation is not suitable for large libraries during a screen. For screening purposes, observing some phenotype (size, color, movement, egg laying) in a way that can be very quickly accessed is ideal. Other ways could include using pathway-specific drug screens using promoter::GFP fusions or utilizing additional *Caenorhabditis* species. Between the dose-dependent response of *C*. *elegans* to the four main anthelmintics and the agreement of the current dead/alive results with past work [37], this study shows that by examining the relative health of *C*. *elegans* as opposed to basic motility, *C*. *elegans* has the potential to be a great model for anthelmintic drug study.

## Supporting information

S1 Fig LT_50_, IT_50_, and IC_50_ for PYR. Graphical representations of the LT_50_ (purple) and IT_50_ (orange) values of increasing concentrations of Pyrantel on N2 *C*. *elegans* (A-F). Graphs correspond to values in Table 2. Graphs obtained through analysis with GraphPad Prism. Graphical representations of the IC_50_ values on day 4 of Pyrantel on N2 *C*. *elegans*. The mean values (of the raw data) with SEM error bars are included. (G) corresponds to the 1–0 scoring value in Table 3 and (H) corresponds to the 3–0 scoring value in Table 3. Graphs obtained through analysis with GraphPad Prism.
(TIF)

S2 Fig LT_50_, IT_50_, and IC_50_ for NTZ. Graphical representations of the LT_50_ (purple) and IT_50_ (orange) values of increasing concentrations of Nitazoxanide on N2 *C*. *elegans* (A-F). Graphs correspond to values in Table 2. Graphs obtained through analysis with GraphPad Prism. Graphical representations of the IC_50_ values on day 4 of Nitazoxanide on N2 *C*. *elegans*. The mean values (of the raw data) with SEM error bars are included. (G) corresponds to the 1–0 scoring value in Table 3 and (H) corresponds to the 3–0 scoring value in Table 3. Graphs obtained through analysis with GraphPad Prism.
(TIF)

S3 Fig LT_50_, IT_50_, and IC_50_ for IVM. Graphical representations of the LT_50_ (purple) and IT_50_ (orange) values of increasing concentrations of Ivermectin on N2 *C*. *elegans* (A-F). Graphs correspond to values in Table 2. Graphs obtained through analysis with GraphPad Prism. Graphical representations of the IC_50_ values on day 4 of Ivermectin on N2 *C*. *elegans*. The mean values (of the raw data) with SEM error bars are included. (G) corresponds to the 1–0 scoring value in Table 3 and (H)corresponds to the 3–0 scoring value in Table 3. Graphs obtained through analysis with GraphPad Prism.
(TIF)

S4 Fig LT_50_, IT_50_, and IC_50_ for ALB on N2. Graphical representations of the LT_50_ (purple) and IT_50_ (orange) values of increasing concentrations of Albendazole on N2 *C*. *elegans* (A-F). Graphs correspond to values in Table 2. Graphs obtained through analysis with GraphPad Prism. Graphical representations of the IC_50_ values on day 4 of Albendazole on N2 *C*. *elegans*. The mean values (of the raw data) with SEM error bars are included. (G) corresponds to the 1–0 scoring value in Table 3 and (H)corresponds to the 3–0 scoring value in Table 3. Graphs obtained through analysis with GraphPad Prism.
(TIF)

S5 Fig LT_50_, IT_50_, and IC_50_ for ALB on Hawaii (CB4856). Graphical representations of the LT_50_ (purple) and IT_50_ (orange) values of increasing concentrations of Albendazole on Hawaii *C*. *elegans* (A-F). Graphs correspond to values in Table 2. Graphs obtained through analysis with GraphPad Prism. Graphical representations of the IC_50_ values on day 4 of Albendazole on Hawaii (CB4856) *C*. *elegans*. The mean values (of the raw data) with SEM error bars are included. (G) corresponds to the 1–0 scoring value in Table 3 and (H) corresponds to the 3–0 scoring value in Table 3. Graphs obtained through analysis with GraphPad Prism.
(TIF)
